# Gene disruption of the DNA topoisomerase IB small subunit induces a non-viable phenotype in the hemoflagellate *Leishmania major*

**DOI:** 10.1186/1471-2180-8-113

**Published:** 2008-07-08

**Authors:** Rafael Balaña-Fouce, Carlos García-Estrada, Yolanda Pérez-Pertejo, Rosa M Reguera

**Affiliations:** 1 Departamento de Farmacología y Toxicología (INTOXCAL), Universidad de León, Campus de Vegazana s/n; 24071 León, Spain

## Abstract

**Background:**

The unusual heterodimeric leishmanial DNA topoisomerase IB consists of a large subunit containing the phylogenetically conserved "core" domain, and a small subunit harboring the C-terminal region with the characteristic tyrosine residue in the active site. RNAi silencing of any of both protomers induces a non-viable phenotype in the hemoflagelate *Trypanosoma brucei*. Unfortunately, this approach is not suitable in *Leishmania *where gene replacement with an antibiotic marker is the only approach to generate lack-of-function mutants. In this work, we have successfully generated null mutants in the small subunit of the *L. major *DNA topoisomerase IB using two selection markers, each conferring resistance to hygromycin B and puromycin, respectively.

**Results:**

We have successfully replaced both *topS *loci with two selection markers. However, to achieve the second transfection round, we have had to rescue the null-homozygous with an episomal vector carrying the *Leishmania major topS *gene. Phenotypic characterization of the *L. major *rescued strain and a *L. major *strain, which co-overexpresses both subunits, shows few differences in DNA relaxation and camptothecin cytotoxicity when it was compared to the wild-type strain. Studies on phosphatidylserine externalization show a poor incidence of camptothecin-induced programmed cell death in *L. major*, but an effective cell-cycle arrest occurs within the first 24 h. S-Phase delay and G_2_/M reversible arrest was the main outcome at lower concentrations, but irreversible G_2 _arrest was detected at higher camptothecin pressure.

**Conclusion:**

Results obtained in this work evidence the essentiality of the *topS *gene encoding the *L. major *DNA topoisomerase IB small subunit. Reversibility of the camptothecin effect points to the existence of effective checkpoint mechanisms in *Leishmania *parasites.

## Background

*Leishmania major *is the aetiological agent of cutaneous leishmaniasis, a zoonotic neglected tropical disease characterized by the presence of ulcerative skin lesions at the mosquito's bite-place [1]. The existing first-line therapies based on pentavalent antimonium salts are antiquated and toxic [2]. Paromomycin-based ointments and triazole antifungal agents (fluconazole, itrakonazole and ketoconazole) have variable and limited efficacy [3] whereas, the clinical trials carried out with the promising alkyl-phospholipid miltefosine [4] are scarce. A very recent report carried out in Iran concluded that oral miltefosine was apparently as good as pentavalent antimonium salts for the treatment of *L. major *cutaneous leishmaniasis [5].

In this scenario there is an urgent need for new antileishmanial drug targets. DNA topoisomerases (Top) catalyze changes in the superhelicity of duplex DNA during replication, transcription, recombination and DNA repair processes [6,7]. Two families and two subfamilies of DNA topoisomerases play a pivotal role preserving DNA integrity in all living organisms. Type I Top (TopI) are ATP-independent monomeric enzymes introducing transient single-stranded breaks in DNA, followed by passage and rejoining. Type II Top (TopII) are multimeric ATP hydrolyzing proteins that generate temporary double-stranded breaks in the double helix, followed by passage and rejoining. TopII not only relaxes positively supercoiled DNA, but also displays catenation/decatenation and knotting/unknotting activities. TopIA subfamily produces transient covalent bonds at the 5' end of the broken DNA, whereas TopIB subfamily has a specific cleavage polarity at the 3' end [8].

Unlike all the organisms studied at present, TopIB from trypanosomes and leishmanias are heterodimeric enzymes [9-11]. Genetic analyses identified a gene, located on the *L. donovani *chromosome 34, which encodes a large subunit (LdTopIL) with an estimated molecular mass of 73 kDa. This protein contains both the non-conserved N-terminus as well as the conserved central core domain of the enzyme, which includes all the residues that interact with DNA, except the DNA-cleaving tyrosine. On the other hand, the small subunit (LdTopIS) is encoded by a second ORF located at chromosome 4. This ORF encodes a 262-long polypeptide with a predicted molecular mass of 28-kDa. This polypeptide contains the phylogenetically conserved "SKxxY" motif, which includes the Tyr-222 that plays a role in DNA cleavage. [12]. This genomic organization was confirmed soon after in African trypanosomes [13] and two genes encoding for each TopIB protomer are annotated into the *T. cruzi *Genome Project [14].

As in most eukaryotic cells, TopIB and TopII are essential to cell life [15,16]. Enzyme silencing of *T. brucei *TopII by small RNA interference (RNAi) produces a singular phenotype lacking kDNA, called dyskinetoplasticy that leads to cell death [17]. Furthermore, RNAi-mediated silencing of gene expression of each subunit of TopIB results in a drastic reduction of both DNA and RNA synthesis in African trypanosomes, mimicking the inhibition of nucleic acid biosynthesis observed when bloodstream trypanosomes are treated with the specific inhibitor camptothecin (CPT) [18].

Inhibitors of DNA topoisomerases represent a major class of anti-cancer drugs and a growing number of them are in clinical use [19]. Several studies have shown that CPT has strong anti-trypanosomal and anti-leishmanial activities *in vitro*, inducing DNA-cleavable complexes at the sub-micromolar range [20]. The outcome of stable DNA cleavage is the generation of single- or double-stranded breaks, which are believed to cause point mutations, fragmentation of the genome and eventually programmed cell death (PCD) [21].

Since the mechanism for RNAi-mediated gene attenuation is not functional in *Leishmania *parasites [22], the generation of null-mutants is only feasible through gene replacement techniques that warranty the total disruption of the target gene. The present work describes the phenotype of a null-mutant in the small subunit of the *L. major *TopIB, its sensitivity to CPT as well as the PCD induced by this inhibitor in the aetiological agent of cutaneous leishmaniasis *L. major*.

## Results

### Double targeted gene replacement of *LmTopS*

**Figure 1:**
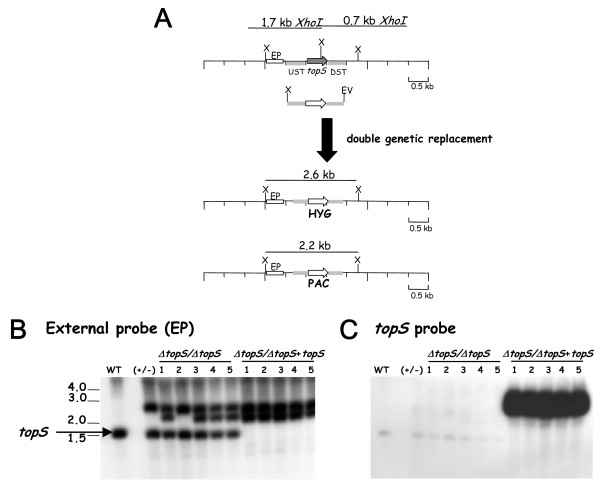
Construction of *L. major topS*-null mutants (*ΔtopS::HYG/ΔtopS::PAC*), by double targeted gene replacement. **A) **Strategy for targeted gene replacement. (Upper) The *topS *gene locus, the location of the upstream (UST) and downstream (DST) segments used to target homologous recombination and 1.7-kb *XbaI *restriction fragments. The disruption constructs are shown immediately below, including the UST and DST fragments, the *XbaI *and *EcoRV *terminal polylinker restriction sites. The targeted gene replacement event is indicated below the thick arrow, showing the structure of the resulting chromosomal locus and the predicted 2.6-kb and 2.2-kb *XbaI *restriction fragments that are diagnostic of the correct homologous integration events. X, indicate *XbaI *restriction sites. Generation of the null-mutant required a second targeted gene replacement using a similar gene disruption cassette containing a PAC marker. **B) **Southern blot containing 10 μg of genomic DNA from WT parasites, heterozygous knockout line after integration of the HYG gene disruption construct (+/-), (*ΔtopS/ΔtopS*) after integration of the PAC, and (*ΔtopS/ΔtopS+topS*)after integration of the PAC in a clone previously transfected with an episomal vector pXG-*topS *carrying the *topS *genehybridized with a radiolabeled probe represented by a white box and located outside of the disruption region (external probe, EP). **C) **The same blot shown in B after elution of the EP and rehybridization to the *topS *probe. The numbers indicate the positions and sizes (kb pairs) of DNA molecular weight markers.

To asses the biological involvement of LmTopIB in relaxation of supercoiled DNA and CPT susceptibility, we tried to create a null-mutant knockout in the *topS *locus by double-targeted replacement with antibiotic resistant cassettes [23], which were kindly provided by S.M. Beverley (University of Washington at St. Louis, Mo USA). Since *topS *gene encodes the catalytic active site of LmTopIB, the effective disruption of this gene should nullify the biological function of the entire enzyme. To that purpose, we created the targeting plasmids pSK-*topS*-KO-HYG and pSK-*topS*-KO-PAC. They contain the respective antibiotic resistance cassettes flanked by a 1000-bp region that includes both the 5' and the 3' flanking regions of the topS gene (Fig. 1A). First, *topS*-allele was replaced by HYG-resistance cassette to create the heterozygous strain (*ΔtopS::HYG*). Hybridization with the external probe (EP) shows a 1.7-kb *XhoI *fragment present in the genomic DNA from WT promastigotes and an additional 2.6-kb fragment corresponding to the effective first allele replacement. Colonies isolated from this heterozygous strain were perfectly viable and were used to perform the second replacement round. When this strain was electroporated with lineal pSK-*topS*-KO-PAC to create the homozygous (*ΔtopS::HYG/ΔtopS::PAC*), a set of double resistant HYG/PAC colonies were obtained (clones 1 to 5), which were able to growth under antibiotics selective pressure. Southern analysis of the isolated clones shows that besides the predicted 2.6 kb corresponding to the first allele replacement, a second 2.2 kb-long band corresponding to the second gene substitution, was present (Fig. 1B). However, the 1.7-kb XhoI fragment, which hybridizes with the EP, still remains, showing an unexpected trisomy in the *topS *locus in clones 1, 3, 4 and 5. To remove this band, the heterozygous (*ΔtopS::HYG*) clone was transfected with the *pXG-topS *construct, an episomal plasmid which expresses the topS through the presence of the DHFR/TS locus of *L. major*. This new strain was used for the second replacement. Under these circumstances the 1.7-kb band effectively disappeared from the isolated clones showing only the 2.2 and 2.6-kb expected bands. They correspond to the correct homologous integrations upstream and downstream of the *topS *locus. A Southern analysis of the clones obtained in these experiments using the whole *topS *gene as a probe, shows the effective replacement of both *topS *alleles (lanes 8 to 12 of Figure 1C), in addition to the very intense 3 kb band corresponding to the episomal *topS *gene required to complement the topoisomerase deficiency. One clone representing this new *L. major *strain was called (*ΔtopS/ΔtopS*) + *topS *indicating the rescued null-*topS *genotype.

**Figure 2:**
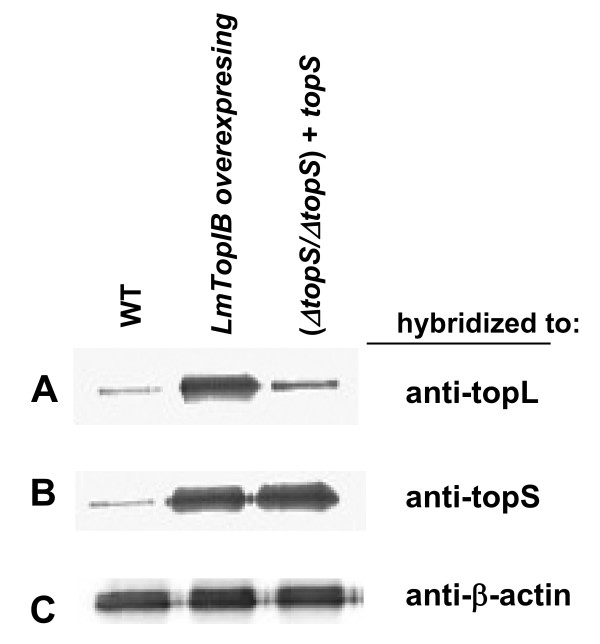
Expression of LmTopIB under different genetic manipulations **A) **Western analysis measuring TopIL protein abundance of WT, LmTopIB-overexpressing and (Δ*topS*/Δ*topS*) + *topS *rescued strain, using a heterologous LdTopIL polyclonal antiserum **B) **Similar to A but hybridizing to a heterologous LdTopIS polyclonal antiserum **C) **Similar to A and B but hybridizing to a heterologous β-actin polyclonal antiserum.

Western analyses from WT, (*ΔtopS/ΔtopS*) + *topS *and LmTopIB overexpressing strains were carried out to assess the expression rate of the TopIB genes. For this purpose, heterologous LdTopIL and LdTopIS polyclonal rabbit antisera were used to hybridize with the transferred proteins after SDS-PAGE. Figure 2A shows the results obtained after hybridization with LdTopIL antiserum, which evidences a significant 7-fold increase of the immunoreactive band corresponding to the LmTopIB-overexpressing strain. Figure 2B shows a similar Western blot, but in this case the nylon membrane was hybridized with LdTopIS rabbit antiserum, showing a *ca*. 7-fold overexpression of the small subunit, in both LmTopIB-overexpressing and (*ΔtopS/ΔtopS*) + *topS *extracts.

### Phenotypic characterization of genetically manipulated *L. major *strains

**Figure 3:**
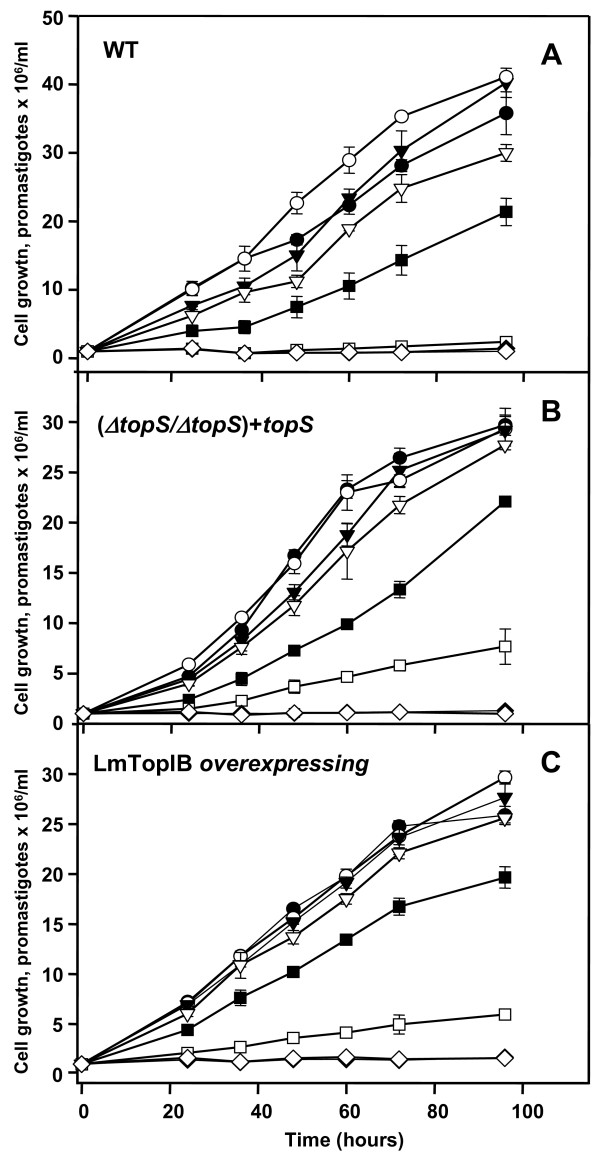
Sensibility of *L. major *WT **(A)**, the rescued (*ΔtopS/ΔtopS+topS*) strain **(B) **and the LmTopIB overexpresing strain **(C) **to the specific TopIB inhibitor CPT. Cell density was monitored at growing concentrations: (○) control; (▼) 0.125 μM; (●) 0.25 μM; (▽) 0.5 μM; (■) 1 μM; (□) 5 μM and -25 (◇) μM CPT by Coulter. Experiments were carried out by triplicate and error bars represent standard deviations.

The phenotypic outcome of genetic manipulation on *topS *locus was evaluated in the rescued (*ΔtopS/ΔtopS*) + *topS*, LmTopIB overexpressing and WT *L. major *strains. The growth rate of these strains was analyzed during a 96 h span-time, 12 h stepwise, in the presence of different concentrations of CPT (0.5 to 25 μM). The proliferation rate was measured by cell counting (Fig. 3A–C) using a Coulter apparatus. No significant differences in growth rate were noticed among the three strains under study. The higher growth found in the WT promastigotes may be due either to the genetic manipulation exerted over the other two strains, or more probably to the continuous antibiotic selection pressure exerted in the culture medium to maintain the strains. CPT was equally effective on the three strains; dose-response curves determined after 48 h subpassages provided IC_50 _values of 0.56 μM, 0.39 μM and 0.30 μM for WT, (*ΔtopS/ΔtopS*) + *topS *and LmTopIB overexpressing strains, respectively.

**Figure 4:**
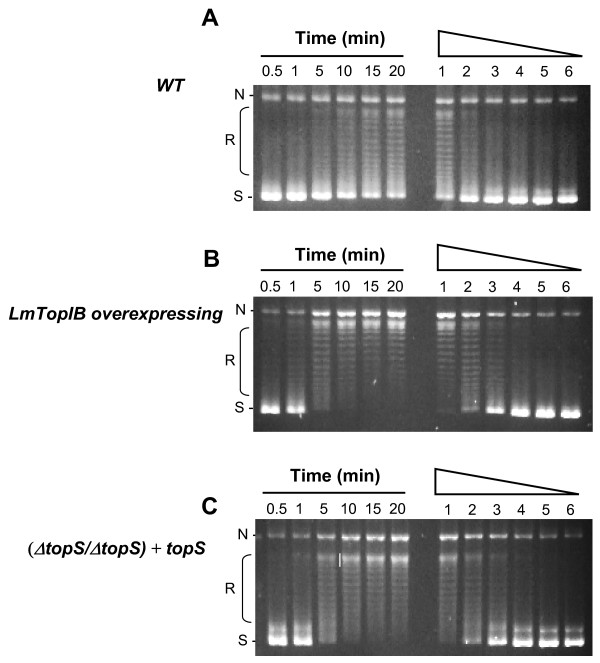
DNA relaxation activity of the *L. major *WT and the strain genetically manipulated at the *topS *locus. 0.2 Micrograms of leishmanial extracts were incubated with 0.125 μg of RfI ΦX174 supercoiled DNA for 30 min at 37°C. Lanes 1 to 6 correspond to time-course experiments (0.5, 1, 5, 10, 15, 20 min). Lanes 7 to 12 correspond to a two-fold serial dilution assay of leishmanial extracts. **A) **WT; **B) **LmTopIB-overexpresing strain; **C) **(*ΔtopS/ΔtopS*) *+ topS *strain; N, nicked plasmid; R, relaxed plasmids; S, supercoiled plasmids.

**Figure 5:**
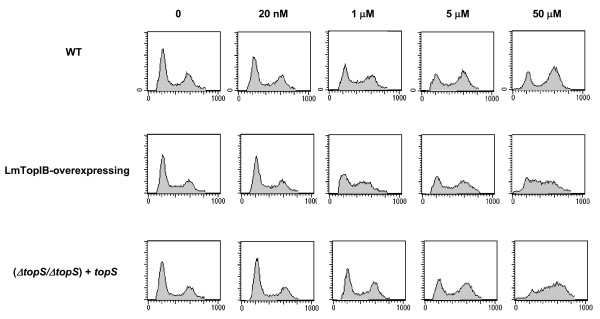
FACS analysis of cell cycle progression and CPT-induced growth arrest in *L. major *promastigotes. Dose-response effect of CPT-inducing cellular arrest. Fluorescence intensity representing the cellular DNA content is shown on the X axis, and cell count is shown on the Y axis. A total of 20,000 events were collected in FACS analysis. The left-hand peak in each panel represents G_1 _cells, the right-hand peak represents G_2 _cells and the points in between, represent S-phase cells. Exponential growing cultures were treated with the indicated concentrations of CPT for 24 h (T24). After drug removal (by rinsing the cultures in drug-free medium), cells were further incubated in fresh medium for 24 hours (R24) or 48 hours (R48). Cells were collected at each time point, and DNA content was analyzed.

To evaluate the topoisomerase activity displayed by these leishmanial strains, standard relaxation assays were performed with WT, (*ΔtopS/ΔtopS*) + *topS *and LmTopIB overexpressing lysates coming from log-phase cultures. Time-course (left lanes) and lysate dilution (right lanes) experiments were carried out at 37°C. Under these experimental conditions a clear distributive relaxation pattern was obtained (Fig. 5) in the three leishmanial strains. The WT strain showed the slower and less active relaxation activity (Fig. 4A). Most of the substrate was relaxed to different topoisomers but perceptible supercoiled DNA still remained after 20 min at the higher protein concentration. The relaxation activity of the other two strains was faster and higher than that shown by the control. Figure 4B shows a fully relaxed DNA after 5 min in the LmTopIB-overexpressing strain, and a 4-fold higher activity (comparing lane 3 in set B with lane 1 in set A). When the LmTopIB-overexpressing strain was compared with the rescued (*ΔtopS/ΔtopS*) + *topS *knockout (Fig. 4C), no differences in both time and protein dilution were observed. It is remarkable that the scarce differences in relaxation activity among strains were in accordance with the lack of significant differences in CPT cytotoxicity.

To determine the phase of the cell cycle at which CPT exerts its growth inhibitory effect, exponentially growing *L. major *promastigotes were treated with different concentrations of CPT for 24 h and analyzed by flow cytometry. Submicromolar concentrations of CPT did not appear to exert any effect. A significant 51% of S-phase arrest appeared after treatment with 1 μM CPT in the (*ΔtopS/ΔtopS*) + *topS *strain, which was increased up to 64% in the presence of 50 μM CPT (Fig. 5).

**Figure 6:**
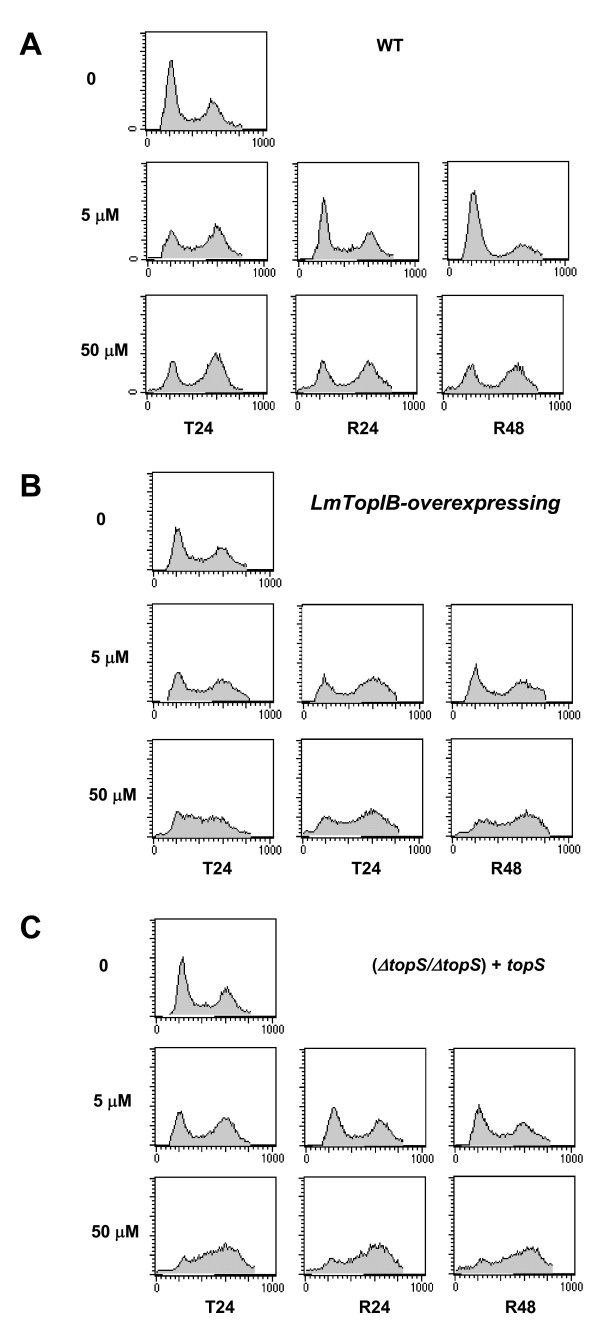
The same experiment to that shown in figure 5 was performed. **B) **WT; **C) **LmTopIB-overexpressing strain and **D) **rescued (*ΔtopS/ΔtopS*) *+ topS *strain. Cells were collected at each time point, and DNA content was analyzed.

To determine whether or not CPT-induced S-phase arrest is reversible, *L. major *promastigotes were treated for 24 h to induce S-phase arrest. Then CPT was washed out and cells were further incubated in fresh medium for an additional period of 24 h (R24) or 48 h (R48). CPT-treated WT cells were able to revert the cell-growth arrest at the lower drug concentration (5 μM) after 24 h, but the arrest was irreversible at 50 μM CPT even after 48 h of drug withdrawal (Fig. 6A). In case of LmTopIB-overexpressing and rescued-mutant (*ΔtopS/ΔtopS*) + *topS *strains, CPT induced irreversible S-phase arrest at any concentration tested (Figs. 6B and 6C).

**Figure 7:**
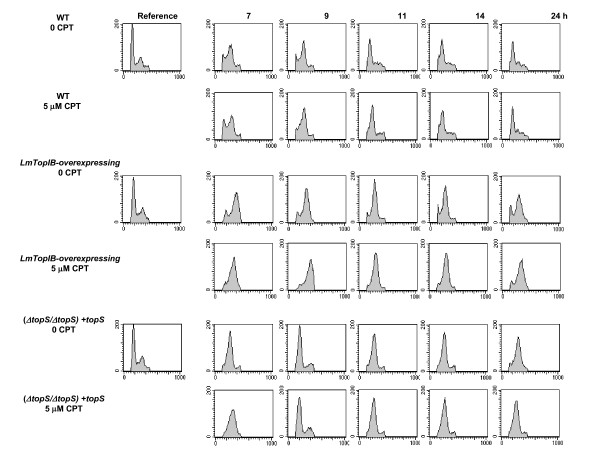
Analysis of DNA content in *L. major *promastigotes after reversible arrest with HU. Cells obtained from 12 h-arrest with 5 mM HU (time 0) were harvested and incubated in fresh media in presence or absence of 5 μM CPT. After the time periods indicated, cells were stained with propidium iodide (PI) and analyzed by flow-cytometry. The graphs represent the relative PI fluorescence plotted against the frequency of events per channel. Each plot represents data collected from 20,000 events/sample.

Progression of the cell cycle after chemical synchronization was studied in the three *L. major *promastigotes strains. The use of 5 mM HU, an inhibitor of ribonucleotide reductase, to reversibly arrest DNA synthesis, has been previously reported [24]. After a 12-h exposure to HU, the percentages of *L. major *promastigotes in the different cell cycle phases were: 65% in G_1_, 23% in S and 12% in G_2_/M. Promastigotes underwent cell cycle progression after HU removal, being the time required to complete the first cell cycle dependent on the genetic manipulation carried out into *topS *locus. Thus, the WT phenotype needs 24 h to complete a cell cycle; this time was longer for the LmTopIB-overexpressing strain, whereas the rescued-mutant (*ΔtopS/ΔtopS*) + *topS *completed the cell-cycle progression in a shorter period of 9 h. These effects were independent of CPT treatment, which strongly suggest, that they were due to the genetic manipulation of the strains (Fig. 7).

### Quantification of PS externalization in *L. major *promastigotes

**Figure 8:**
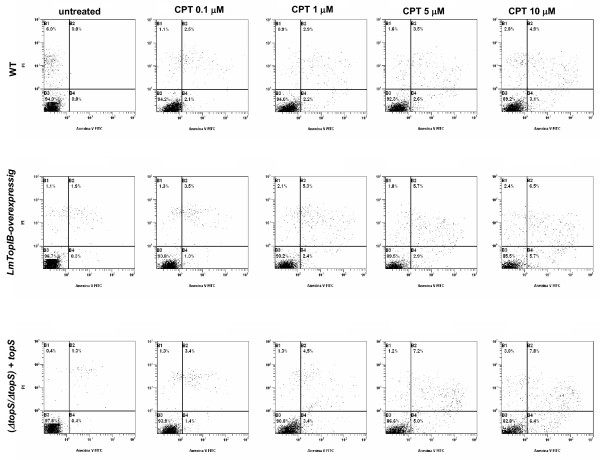
PS externalization as a consequence of CPT exposure; WT, LmTopIB-overexpressing and rescued (*ΔtopS/ΔtopS*)*+topS *strains were treated with different concentrations of CPT for 24 h and analyzed for PCD. Dead cells were excluded by PI incorporation. Dot plots are representative of three independent assays.

**Figure 9:**
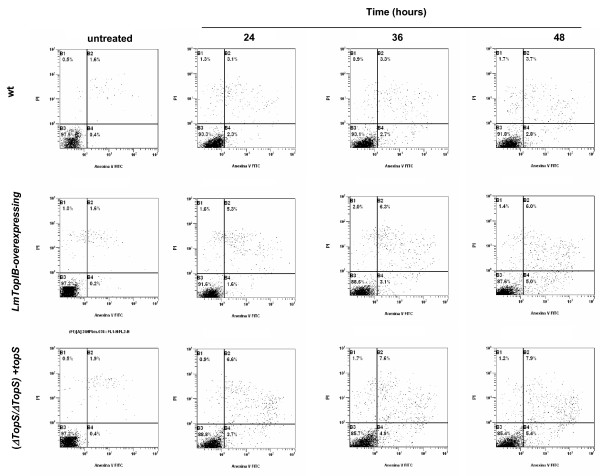
Time-course experiment of PS externalization due to CPT exposure. WT, LmTopIB-overexpressing and rescued (*ΔtopS/ΔtopS*)*+topS *strains were exposed to 5 μM CPT treatment for 24, 36 and 48 h. PS externalization was monitored by FACS. Dead cells were excluded by PI incorporation. Dot plots are representative of three independent assays.

As a measure of CPT-induced PCD in *L. major*, a FACS procedure was carried out combining PI/annexin V double stain. Promastigotes were cultured in presence of different concentrations of CPT and at different time-points to find the optimal conditions for PCD. Figure 8 shows the translocation of PS to the outer surface of plasma membrane of drug-treated promastigotes at 0.1 μM, 0.5 μM, 1 μM, 5 μM and 10 μM CPT. The three *L. major *strains used in the study were incubated with different CPT concentrations during a period of 24 h and then they were submitted to the double stain protocol. Panels show a slight increase of early and late apoptosis (bottom and upper-right sections of each panel, respectively) regarding the untreated cultures, which barely rose with CPT concentration. This effect was also studied in the (*ΔtopS/ΔtopS*) + *topS *and LmTopIB-overexpressing strains, which scarcely increased this parameter. Since CPT did not significantly induce PCD at 24 h, we extended the incubation period up to 48 h using 10 μM CPT. Apoptosis was assessed after this time (Fig. 9). Surprisingly, no significant differences were observed in the three strains. These results indicate that CPT is not inducing PCD in *L. major *cultures under the experimental conditions using in our lab.

## Discussion

Double targeted gene replacement is the method of choice to create defective null-mutants in *Leishmania *parasites [22]. A *L. major *TopIB-null mutant defective in the small subunit of this enzyme (which contains the DNA-cleaving tyrosine) was created to assess the role played by this enzyme in parasite survival and CPT resistance. Since no colonies were obtained repeatedly after a second round of replacement, we suspected this gene was essential to *Leishmania *and its activity was not made up for other Top enzymes. The outcome of incomplete replacement of both LmTopIB alleles by selectable antibiotics was the emergence of trisomies for the *topS *locus (lanes 1, 3, 4 and 5, Fig. 1), which is a non-atypical phenomenon in kinetoplastids because of the plasticity of their genome [25]. Therefore, we decided to perform a previous transformation with a plasmid carrying the target gene for episomal expression and genetic complementation before the second round of genomic replacement. In this case, we successfully obtained several colonies, in which the *topS *allele was replaced by the antibiotic marker. Essentiality of TopIB has been described in *Drosophila *[15] and mouse [16], where the outcome of TopIB gene disruption is a dead phenotype. The budding yeast is a remarkable exception to this phenomenon [26]. *S. cerevisiae*-deficient TopIB is viable and it is currently used as an important tool for gene expression of foreign recombinant TopIB, because the expression of these enzymes in bacteria is difficult and sometimes not feasible.

Gene silencing of *TbTopIL *and *TbTopIS *genes was carried out by Bakshi and Shapiro [18] using RNAi. These authors found indistinct phenotypes when separately silenced *TbTopIL *or *TbTopIS *genes; detectable reduction in ribosomal RNA as well as in levels of specific messengers, growth arrest, decrease in total nucleic acid biosynthesis attributable to reductions in both DNA- and RNA-specific synthesis. Absolute levels of nuclear and mitochondrial transcripts were reduced. In *L. major *the single replacement of one of the alleles originated a heterozygous phenotype that was no differentiable from the WT in both cell growth and CPT sensitivity.

Neither the promastigotes from the genetically rescued *topS*-null mutant nor the ones from TopIB overexpressing strain, showed different growth rates or significant IC_50 _values to CPT. These unexpected results correlated well with the relaxation activities found in the extracts obtained from the different strains, where scarce differences were accounted. One likely explanation to this finding is the possible degradation of the overexpressed subunit, unless it is assembled with the large subunit building an active enzyme. There are evidences showing that a coordinated expression of both subunits is necessary in African trypanosomes. RNAi silencing of any one of the TbTopIB protomers causes coordinated loss of both subunits [18]. The authors have speculated that individual proteins are stable only in association with each other, becoming unstable when separated.

Several reports conclude that TopIB overexpression does not involve higher relaxation activity or CPT susceptibility in mammalian cells [27]. A recent paper describing the transfection of human TopIB gene into OVCAR-3 cultures – an ovarian cancer cell line – shows that, despite an effective seven-fold TopIB overexpression, the sensitivity to topotecan – a CPT hydrosoluble analogue – was not improved compared with control untransfected cells [28]. This observation agrees with the similar levels of TopIB activity found in control and overexpressing cells, pointing to an effective post-translational down-regulation mechanism that is limiting TopIB activity to supportable values. These authors were tempted to speculate that TopIB overexpression produces an alteration in the phosphorylation state of the protein that limits its activity and prevents improvement of CPT response [29,30]. It is very likely that the dispensable 40 amino acids-long sequence comprised between Ser-96 and Ser-135 within the N-terminal extension of LmTopIB small subunit which contains 27 serine residues [31,32], may be suitable of phosphorylation/dephosphorylation processes thus acting as a post-translational switch mechanism.

CPT toxicity depends on the ability of DNA repair systems to restore the basic cellular functions. TopIB overexpression may trigger the repair mechanisms linked to this enzyme such as tyrosyl diphosphodiesterase I (TdpI), which prevents the formation of ternary complexes with DNA in presence of CPT as well as the ubiquitination/sumoylation pathways [33]. Ubiquitination is a down-regulation mechanism that takes place with the hyperphosphorylated enzyme. TopIB degradation by 26S proteasome may be useful to increase tolerance to DNA-cleaving poisons, facilitating the phosphodiesterase activity of Tdp1 to excise Top1 from cleaved DNA [34]. On the other hand, sumoylation seems to be a stimulatory system via relocation of TopIB from nucleoplasm to the nucleolus preventing the ubiquitin-mediated enzyme degradation [35].

Flow-cytometric analysis of 12 h HU-arrested *L. major *promastigotes shows a different cell-cycle recovering pattern after drug withdrawal. The LmTopIB-overexpressing strain shows a S-phase delay of the cell cycle, which is not fully completed 24 h after chemical arrest. On the contrary, the rescued-mutant (Δ*topS*/Δ*topS*) + *topS *completes the cell cycle in a shorter period of 9 h, three hours before the WT. Synchronized *L. major *promastigotes were exposed to different concentrations of CPT for a period of 24 h and then they were washed and incubated up to 48 h in a CPT-free medium. No differences were detected amongst the distinct strains at CPT concentrations below submicromolar range. However, 1 μM CPT produced S-phase delay in WT and (Δ*topS*/Δ*topS*) + *topS *strains and reversible G2 arrest at 5 μM CPT. The constant pressure (up to 24 h) following CPT removal induces the activation of an S-phase checkpoint in mammalian cells. Checkpoint activation provides additional time for DNA repair before starting a new cell cycle [36]. Twenty four hours after 5 μM CPT removal results in a complete recovery of the cell cycle in WT promastigotes, but a large percentage of (*ΔtopS/ΔtopS*) + *topS *and LmTopIB-overexpressing promastigotes still remained arrested at G_2_/M phase. However, 50 μM CPT produced a clear irreversible arrest in G_2_/M in the three strains analyzed.

Like other DNA damaging agents, CPT is an efficient inducer of PCD [37,38]. Sen and coworkers [39] have described that CPT-mediated leishmanicidal effect in *L. donovani *promastigotes appears after the mitochondrial function is inhibited, which is in turn followed by an increase in mitochondrial membrane potential. These authors showed that CPT increases the intracellular concentration of reactive oxygen species (ROS), with the concomitant rise in lipid peroxidation and decline in the concentration of the free radical scavenger glutathione. In a second report, these authors described the involvement of caspase-3 and poly-(ADP-ribose) polymerase (PARP) cleavage in CPT-mediated apoptosis after depletion of intracellular K^+ ^and DNA fragmentation, in the same time span [40]. These results were no reproducible in *L. major *promastigotes under the assay conditions expressed in Materials and Methods. Expanded time points and concentrations were not able to produce the effects described by these authors. Unlike the aforementioned results, where more than 50% apoptosis was found after an early 4-h exposure to 5 μM CPT, we could only detect – using the PS/PI double stain assay – a scant 12% apoptosis after 48 h exposure to CPT. According to the current knowledge on trypanosomatids genome, the apoptosis pathway described by the authors downstream CPT-induced ROS production, is very unlikely. No genes involved in the caspase cascade and PARP cleavage have been annotated in the *Leishmania *genome Project and the metacaspase-like proteins recently described are not responsible for the caspase-like activities in PCD [37,41]. Time and concentration experiments showed that the episomal overexpression of the complete LmTopIB had poor impact in CPT-mediated *L. major *apoptosis. These results disagree with previous experiments carried out in COS cells transfected with the yeast *topIB *encoding gene (ScTopIB), since CPT treatment resulted in preferential killing via apoptosis of those cells expressing the yeast enzyme rather than in "mock" transfected cells [42]. A possible explanation of these differences might consist in the high differences in relaxation activity (ca. 100-fold) produced by the transient transfection of *ScTopIB *gene in COS cells.

## Conclusion

The *topS *gene is essential for *L. major *survival, since the previous genetic rescue with an episomal plasmid carrying the *topS *gene was necessary to obtain a complete double replacement of *both *topS alleles. Rescued (Δ*topS*/Δ*topS*) + *topS *and LmTopIB-overexpressing strains had no characteristic phenotype in both relaxation activity and CPT resistance. This lack of phenotype can be due to post-translational modifications or because the threshold that may be needed to alter its function was not reached. However, CPT treatment and subsequent removal produced a recovery of the arrested cell-cycle in G_2_/M, which was delayed in the overexpressing strain. The poor CPT-induced PCD observed in the three *L. major *promastigote strains suggests alternative cytotoxic mechanisms other than those previously shown in *L. donovani*.

## Methods

### Parasite cultures

*L. major *LV39c5 promastigotes (WT strain) were cultured at 26°C in M199 supplemented with 10% (v/v) heat-inactivated fetal calf serum (FCS).

### Drug solutions

CPT (Sigma Chemical Co. St. Louis USA) was dissolved in dimethylsulfoxide (DMSO) at 20 mM final concentration and stored at -20°C.

### Cloning of *LdTopS *and *LdTopL *genes and molecular constructs

The gene encoding the catalytic subunit of LmTopIB (*topS *a 789-bp fragment) was amplified by PCR from *L. major *genomic DNA using the primers 5'-tcccccgggccaccATGCAGCCTGTTCAAAGTCCT-3', and 5'-cgcggatcctcaaaaatcgaagttctcggc-3' (capitalized letters are from *topS*). Sequence of these primers was extracted from the *L. major *Genome Database [43]. The resulting fragment was digested with *SmaI *and *BamHI *and cloned into the expression vector pXG [44] to make pXG-*topS*. The integrity of this plasmid was confirmed by sequencing.

Two *topS *deletion constructs were created: pSK-*topS*-KO-HYG and pSK-*topS*-KO-PAC. The *topS *ORF upstream (UST) and downstream (DST) sequences (1.0 kb each) were amplified by PCR using primers that incorporated restriction sites suitable for subsequent insertion into the polylinker region of the pSK Bluescript (Stratagene) vector in a "head-to-tail" manner. The DNA fragments corresponding to hygromycine B (HYG) and puromycin (PAC) selection antibiotics were based on the *Leishmania *expression vector series pXG-HYG and pXG-PAC [45]. HYG and PAC ORFs were obtained by digestion with *BamHI *and *SpeI *and cloned in between the UST and DST sequences of the *topS *ORF. The resulting constructs (pSK-*topS*-KO-HYG, and pSK-*topS*-KO-PAC) were digested with *NotI-EcoRV *to obtain linear *topS *deletion fragments (3.0 kb for HYG-cassette and 2.6 kb for PAC-cassette, respectively).

*LmTopL *encoding gene corresponds to the large DNA-binding subunit of leishmanial TopIB. It was amplified from *L. major *genomic DNA using the oligonucleotides 5'-tcccccgggccaccATGAAGGTGGAGAATAGCAAGATG-3' and 5'-cgcggatccCTACACCCTCAAAGCTGCAAGAGG-3' (capitalized letters are from *topL*). The resulting fragment was digested with *SmaI-BamHI *and cloned into the expression vector pXG-HYG, thus constituting the plasmid pXG-HYG-*topL*. An overexpressing LmTopIB strain was obtained by double transformation with pXG-*topS *and pXG-HYG-*topL*.

### Deletion of the *L. major topS *gene

In order to replace first *topS *allele, *L. major *WT promastigotes, were grown up to 5 × 10^6 ^per ml, washed in cold cytomix (120 mM KCl, 0.15 mM CaCl_2_, 10 mM K_2_HPO_4_, 25 mM Hepes pH 7.6, 2 mM EDTA, 5 mM MgCl_2_) [46] and resuspended in the same solution at a concentration of 1 × 10^8 ^promastigotes per ml. Five hundred-microliter aliquots were electroporated twice with 5 μg of the linear 3.0-kb *NotI*-*EcoRV topS*::HYG fragment (1.5 kV, 25 μF using a Bio-Rad Gene Pulser II apparatus) in 0.4 cm electrode gap cuvettes, transferred to 10 ml of M199 plus 10% FCS and incubated at 26°C for 8 h in absence of antibiotics. Cells were spun down, and the pellet was resuspended in 100 μl of fresh M199 plus 10% FCS and plated on semisolid medium containing 30 μg/ml HYG. Heterozygotes showing the replacement of one of the alleles *topS *(+/-) were subjected to the second round of gene disruption. With this purpose, they were electroporated with 5 μg of the 2.6-kb *NotI*-*EcoRV topS*::PAC fragment. Five clones (*topS*-1–5) that grew in the presence of 30 μg/ml HYG and 30 μg/ml PAC were selected as *topS*-null mutants (*ΔtopS::HYG/ΔtopS::PAC*). Successful integration of both cassettes was achieved in four out of the five clones. However, the replacement of the *topS *alleles was incomplete in all the clones.

A new approach to get a double gene replacement was carried out using *topS *heterozygous clones, which were transfected with pXG-*topS*. Those clones that were resistant to 30 μg/ml HYG and 10 μg/ml G418 were selected and named (-/+)/+ *topS*. This new clone was used to perform the second round of gene disruption. Electroporation was performed with 5 μg of the 2.6-kb *NotI-EcoRV topS*::PAC fragment. Five clones that grew in the presence of 30 μg/ml HYG, 10 μg/ml G418 and 30 μg/ml PAC were selected as rescued *topS*-null mutants (*ΔtopS::HYG/ΔtopS::PAC*) + *topS*, (this strain will be called: (*ΔtopS/ΔtopS*) + *topS *from now on), since they have the episomal plasmid pXG-*topS*.

### SDS-PAGE and Western Blotting

*L. major *promastigotes were harvested at different times during the growth and washed twice with PBS. After sonication and centrifugation at 10,000 × g for 20 min, the supernatant was removed. Five micrograms of protein from each time point were diluted in the loading buffer (60 mM Tris-HCl, pH 6.8, 2% SDS, 5% 2-mercaptoethanol, and 5% glycerol), boiled for 5 min, and analyzed by SDS-PAGE (12% acrylamide, 2.7% bisacrylamide). Proteins were electrotransferred onto PVDF membranes (Sigma) for 12 h at 25–30 V/cm, and the blots were blocked by incubation in 10 mM Tris-HCl, pH 7.5, 1 M NaCl, 0.5% Tween 20, 5% non-fat milk powder (w/v) for 1 h at room temperature. Polyclonal primary rabbit antibodies against *L. donovani *large LdTopIL, small LdTopIS subunits and β-actin were added to this buffer and the blot was incubated for 2 h. The filter was washed thoroughly in 10 mM Tris-HCl, pH 7.5, 1 M NaCl, 0.5% Tween 20 and then incubated with an anti-rabbit antibody conjugated to horseradish peroxidase (Sigma Chemical Co. St. Louis USA). Antibodies were detected using 3,3'-diamino benzidine as substrate (Biorad).

### Assays for TopIB-mediated relaxation of plasmid DNA

The relaxation activity of LmTopIB was assayed at the indicated time points using 2-fold serial dilutions of leishmanial lysates. Each reaction contained 125 ng of supercoiled close circular DNA from the virus Φ X-174 (Rf I) in 20 μl of reaction buffer (100 mM KCl, 10 mM Tris-HCl, pH 7.5, 1 mM DTT, 1 mM EDTA). The reactions were incubated at 37°C for 20 min and then stopped by the addition of 5 μl of 5 × stop buffer (2.5% SDS, 25 mM EDTA, 25% Ficoll 400, 0.08% bromophenol blue, 0.08% xylene). The products were analyzed by electrophoresis in 1% agarose gel, stained with ethidium bromide, and visualized with a UV transilluminator.

### Flow-cytometric analysis of DNA content

For flow-cytometric analysis, 4 × 10^6 ^promastigotes were harvested by centrifugation at 660 × g, washed twice with PBS, resuspended in 1 ml of fixative solution (30% PBS/70% methanol) and incubated at 4°C for 1 h. Afterwards, parasites were collected by centrifugation, resuspended in PBS containing 20 μg/ml of RNaseA (Roche, Mannheim, Germany) and incubated for 20 min at 37°C. After incubation, the cells were harvested, resuspended in 1 ml of citrate buffer (45 mM MgCl_2_, 30 mM sodium citrate, 20 mM Mops, pH 7.0, 0.1% Triton X-100), and stained by the addition of 50 μg of propidium iodide (PI) (Sigma, St. Louis, MO, U.S.A.) followed by incubation at 37°C for 20 min. Fluorescence was determined by flow cytometry on an FACSCalibur flow cytometer (Becton Dickinson, San Jose, CA, U.S.A.).

### Cell cycle synchronization and CPT treatment

**Figure 10:**
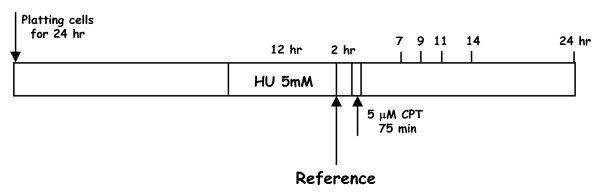
Scheme of the culture synchronization with 5 mM HU and CPT treatment.

For the synchronization of DNA replication, exponentially growing *L. major *promastigotes (5 × 10^6 ^cells/ml) were arrested with 5 mM hydroxyurea (HU) for 12 h. Afterwards parasites were harvested, washed twice with PBS and resuspended in fresh medium for 2 h before use. Cells were then treated with 5 μM CPT for 75 min and finally incubated in a CPT-free medium for the time indicated in the scheme of Figure 10.

### Determination of apoptosis

Apoptosis was detected by translocation of phosphatidyl serine (PS) to the cell surface with the annexin V-FITC reagent (BD Pharmigen). Fraction of annexin V-positive cells was measured with CellQuest software (BD Biosciences, San Jose, CA).

## List of abbreviations

Camptothecin (CPT); dimethylsulfoxide (DMSO); external probe (EP); Fluorescence Activated Cell Sorting (FACS); Fetal Calf Serum (FCS); hydroxyurea (HU); hygromycine B (HYG); large subunit of leishmanial DNA topoisomerase (LmTopIL); small subunit of leishmanial DNA topoisomerase (LmTopIS); puromycin (PAC); Poly-(ADP-ribose)-polymerase (PARP); Programmed Cell Death (PCD); propidium iodide (PI); phosphatidylserine (PS); Small RNA interference (RNAi); Reactive Oxygen Species (ROS); type IB DNA topoisomerase (TopIB); type II DNA topoisomerase (TopII); yeast DNA topoisomerase (ScTopIB); wild-type (WT).

## Authors' contributions

RBF and RMR conceived the study, participated in its design and performed the deletion and complementation of the *LmTopS gene*. YPP and CGE participated in the construction of plasmids and performed the phenotypical analyses. RBF, CGE, YPP and RMR drafted the manuscript and RMR revised the article. All authors read and approved the final manuscript.
